# Real-world utilization of aromatase inhibitors, tamoxifen, and ovarian function suppression in premenopausal patients with early hormone receptor-positive, HER2-negative breast cancer with increased recurrence risk

**DOI:** 10.1016/j.breast.2025.104458

**Published:** 2025-03-22

**Authors:** Volkmar Müller, Manuel Hörner, Marc Thill, Maggie Banys-Paluchowski, Sabine Schmatloch, Peter A. Fasching, Nadia Harbeck, Dagmar Langanke, Sabrina Uhrig, Lothar Häberle, Dorothea Fischer, Alexander Hein, Tanja N. Fehm, Chloë Goossens, Jürgen Terhaag, Uwe Heilenkötter, Peter Dall, Christian Rudlowski, Rachel Wuerstlein, Mustafa Aydogdu, Mignon-Denise Keyver-Paik, Carolin Hammerle, Natalija Deuerling, Elmar Stickeler, Bahriye Aktas, Erik Belleville, Martin Thoma, Nina Ditsch, Yasmin Baila, Christian Roos, Christian Mann, Caterina Iuliano, Sara Y. Brucker, Andreas Schneeweiss, Andreas D. Hartkopf

**Affiliations:** a Department of Gynecology, Hamburg-Eppendorf University Medical Center, Hamburg, Germany; b Department of Gynecology and Obstetrics, Universitätsklinikum Erlangen, Friedrich-Alexander-Universität Erlangen-Nürnberg, Erlangen, Germany; c Comprehensive Cancer Center Erlangen-EMN (CCC ER-EMN), Erlangen, Germany; d Department of Gynecology and Gynecological Oncology, Agaplesion Markus Krankenhaus, Frankfurt, Germany; e Department of Gynecology and Obstetrics, University Hospital Schleswig-Holstein, Campus Lübeck, Lübeck, Germany; f Frauenklinik, Elisabeth-Krankenhaus Kassel, Kassel, Germany; g Breast Center, Department of Gynecology and Obstetrics and CCC Munich LMU, LMU University Hospital, Munich, Germany; h Frauenklinik, St. Elisabeth-Krankenhaus Leipzig, Leipzig, Germany; i Biostatistics Unit, Department of Gynecology and Obstetrics, Universitätsklinikum Erlangen, Erlangen, Germany; j Frauenklinik, Klinikum Ernst von Bergmann, Potsdam, Germany; k Frauenklinik, Klinikum Esslingen GmbH, Esslingen Germany; l Department of Gynecology and Obstetrics, University Hospital Düsseldorf, Center for Integrated Oncology (CIO Aachen, Bonn, Cologne, Düsseldorf), Düsseldorf, Germany; m Department of Gynecology and Obstetrics, Rottal Inn Kliniken, Eggenfelden, Germany; n Klinik für Frauenheilkunde und Geburtshilfe, Klinikum Itzehoe, Itzehoe, Germany; o Frauenklinik, Städtisches Klinikum Lüneburg, Lüneburg, Germany; p Frauenklinik, Evangelisches Krankenhaus Bergisch Gladbach, Bergisch-Gladbach, Germany; q Klinik für Gynäkologie, Gynäkoonkologie und Senologie Klinikum Bremen-Mitte, Bremen, Germany; r Frauenklinik des Klinikums der Stadt Wolfsburg, Wolfsburg, Germany; s Frauenklinik, St. Josefs- Hospital Wiesbaden, Wiesbaden, Germany; t Frauenklinik und Brustzentrum, Klinikum Fichtelgebirge gGmbH, Marktredwitz, Germany; u Department of Obstetrics and Gynecology, Center for Integrated Oncology (CIO Aachen, Bonn, Cologne, Düsseldorf), University Hospital of RWTH Aachen, Aachen, Germany; v Department of Gynecology, University of Leipzig Medical Center, Leipzig, Germany; w ClinSol GmbH & Co KG, Würzburg, Germany; x Brustzentrum, Ammerland-Klinik, Westerstede, Germany; y Department of Gynecology and Obstetrics, University Hospital Augsburg, Augsburg, Germany; z Frauenklinik, Klinikum Kassel, Kassel, Germany; aa Novartis Pharma GmbH, Sophie-Germain-Str. 10, 90443 Nuermberg, Germany; ab Department of Gynecology and Obstetrics, Tübingen University Hospital, Tübingen, Germany; ac National Center for Tumor Diseases, University Hospital and German Cancer Research Center, Heidelberg, Germany

**Keywords:** Early breast cancer, Endocrine therapy, Tamoxifen, Aromatase inhibitor, Hormone receptor-positive

## Abstract

**Background:**

The optimal adjuvant endocrine treatment in premenopausal patients with hormone receptor-positive, HER2-negative (HRpos/HER2neg) early breast cancer (eBC) remains debated, particularly the choice between aromatase inhibitors plus ovarian function suppression (AI + OFS) or tamoxifen (TAM) with or without additional OFS. This study assessed the use of adjuvant endocrine therapies for premenopausal patients with intermediate/high-risk HRpos/HER2neg eBC.

**Methods:**

CLEAR-B (AGO-B-059; NCT05870813) was a retrospective study analyzing data, collected from January 2016 to June 2019 and from January 2022 to December 2023 during the certification process of breast centers in Germany. Premenopausal patients with HRpos/HER2neg intermediate/high-risk eBC were eligible. Patient and disease characteristics, in addition to recommended and received adjuvant treatments, were evaluated.

**Results:**

The number of registered patients was 3137, of whom 2789 had complete information on endocrine treatments (1717 for 2016–2019 and 1072 for 2022–2023). In 2016–2019, 8.4 % of the patients were recommended to be treated with AI + OFS, whereas in 2022–2023, the proportion of patients with a treatment recommendation for AI + OFS rose to 42.1 %. In 2016–2019, TAM monotherapy was most frequently recommended (80.8 %). Conversely, TAM + OFS was not commonly recommended (9.3 % in 2016–2019 and 16.5 % in 2022–2023). While no clear association between tumor stage and chosen endocrine therapy was found in 2016–2019, most patients with ≥stage IIA were recommended to be treated with AI + OFS in 2022–2023.

**Conclusion:**

This analysis shows that treatment recommendation for AI + OFS in premenopausal patients with HRpos/HER2neg eBC increased relevantly in the past years, reflecting latest guideline recommendations.

## Highlights

• Treatment recommendations for patients with HRpos/HER2neg eBC have evolved.
• In Germany in 2016–2019, tamoxifen monotherapy was predominantly recommended.
• In Germany in 2022–2023, aromatase inhibitors + OFS therapy was predominantly recommended.

## 1 Introduction

Early breast cancer (eBC) is the most common cancer among women in Germany, with approximately 74,500 new cases diagnosed annually. The incidence of eBC has increased significantly over the years. Treatment options for patients with hormone receptor-positive, HER2-negative (HRpos/HER2neg) eBC can vary due to tumor biology and different hormonal regulation mechanisms for estradiol production [1,2]. Furthermore, age, especially young age, is a prognostic factor in treatment guidelines [[3], [4], [5]]. Both chemotherapy and the extent of endocrine treatments for patients with HRpos/HER2neg eBC are the subjects of several studies.

Regarding chemotherapy, most recent studies focus on identifying patient populations that may benefit from chemotherapy de-escalation. Studies that include multigene assays for prognostication highlight the importance of considering patient age and menopausal status when deciding on the use of chemotherapy for HRpos/HER2neg eBC (MINDACT, TailorX, RxPONDER [[6], [7], [8]]). These studies show that young patients (<50 years or premenopausal) in the chemotherapy arm have, to some extent, a better prognosis, while for postmenopausal women—even those with an intermediate recurrence risk—chemotherapy does not improve prognosis and can be avoided [[6], [7], [8]]. These studies are the focus of further investigations because the effect of chemotherapy on the prognosis of premenopausal patients may be mediated by the effect of chemotherapy on the ovarian function [[9], [10], [11]]. Therefore, the question remains whether this effect of chemotherapy on prognosis could also be achieved with adequate endocrine therapy including ovarian function suppression (OFS).

In addition to the potential use of chemotherapy, all patients with HRpos/HER2neg eBC should be treated with adjuvant endocrine therapy. Three treatment options are the standard-of-care therapies for this patient population: tamoxifen (TAM) monotherapy, TAM combined with OFS (TAM + OFS), and aromatase inhibitors combined with OFS (AI + OFS). OFS is usually performed with gonadotropin-releasing hormone (GnRH) analogs, although oophorectomy is also used in some cases. Recommendations for adjuvant endocrine therapy for patients with HRpos/HER2neg eBC have varied over the years. Recently, a risk-based strategy has become the most commonly recommended approach in most therapy guidelines [4,[12], [13], [14], [15], [16], [17], [18], [19]]. Premenopausal patients with a lower risk of recurrence are recommended to be treated with TAM, while those with a higher risk of recurrence should be treated with TAM + OFS or AI + OFS. Although AI + OFS is considered the most effective therapy in terms of disease-free survival, it is not typically recommended for all patients due to its unfavorable side effect profile and lack of benefit regarding overall survival [20,21]. In addition to the challenging status quo of the evidence, no consistent definition of low-risk versus high-risk patients exists. Nevertheless, a considerable degree of variability between countries seems evident with regard to the utilization of endocrine therapy, even in patient populations with a higher risk of recurrence. Notably, in the monarchE trial, which evaluated adjuvant treatment with the CDK4/6 inhibitor abemaciclib in combination with endocrine therapy and recruited patients in 2017–2019, the use of AI in premenopausal patients in Germany was 22 % [22]. It has to be noted that for premenopausal patients, German and international guidelines [12] require the addition of GnRH agonists, so it can be assumed that the vast majority was also treated with AI + OFS.

Therefore, the current study aimed to assess the therapeutic approach for adjuvant endocrine therapy for premenopausal patients with HRpos/HER2neg eBC at increased risk of recurrence in a real-world setting in Germany. To this end, the choice of endocrine therapy (TAM monotherapy, TAM + OFS, AI + OFS) was evaluated in relation to patient and disease characteristics in two periods: 2016–2019 and 2022–2023.

## 2 Method

### 2.1 Study and study sites

CLEAR-B (Cancer Landscape - Early Adjuvant Retrospective Registry - Breast Cancer) is a retrospective study of the AGO Breast group (AGO-B-059; NCT05870813), which was conducted in 2023–2024. The study was reviewed and approved by the Ethics Committee of the Medical Faculty of the Friedrich-Alexander-Universität Erlangen-Nürnberg (application number: 23-1-Br, approval date: January 19, 2023). As the data were collected anonymously (all personal information and personal dates were removed), the patients did not have to provide informed consent. The study was carried out following the Good Clinical Practice and Declaration of Helsinki guidelines. The participating study sites were breast cancer centers certified by the German Society of Breast Diseases (Deutsche Gesellschaft für Senologie e.V.) and the German Cancer Society (Deutsche Krebsgesellschaft e.V.). As part of their annually audited certification processes, these centers are required to prospectively document all breast cancer patients in consecutive order in a database, enabling them to generate a complete list of all patients treated. In CLEAR-B, the study sites documented all premenopausal patients with a primary diagnosis in January 2016–June 2019 or January 2022–December 2023. These periods were chosen to match the recruitment period of the NATALEE study (2016–2019) [23,24] and to be able to compare the data to a more recent dataset (2022–2023). A total of 56 study sites across Germany enrolled their patients in CLEAR-B (Supplementary Table 1).

### 2.2 Patient population

Premenopausal patients with HRpos/HER2neg eBC and an increased risk of recurrence could be included. The participating study sites were asked to identify and document all patients with the following inclusion and exclusion criteria: All patients had to be premenopausal. With regard to the risk profile, they had to be treated with chemotherapy before the start of endocrine treatment or have a tumor of ≥2 cm as assessed from the surgery specimen or at least one positive lymph node as assessed at definitive surgery. A complete list of inclusion and exclusion criteria is shown in Supplementary Table 2.

### 2.3 Data collection

Before enrolling patients in CLEAR-B, the study sites were asked to check the completeness of all requested data and ensure that a follow-up of recurrence status and survival status was available. Patient and disease characteristics were documented in a dedicated electronic case report form and anonymized after the study site indicated that the data were complete. An overview of the documented patient and disease characteristics is shown in Supplementary Table 3. The recommended therapy choices were documented as “intention-to-treat” variables (the therapies recommended by the breast cancer centers’ multidisciplinary tumor boards), and the received therapies were documented as “per-treatment” variables.

### 2.4 Study populations

**Fig. 1:**
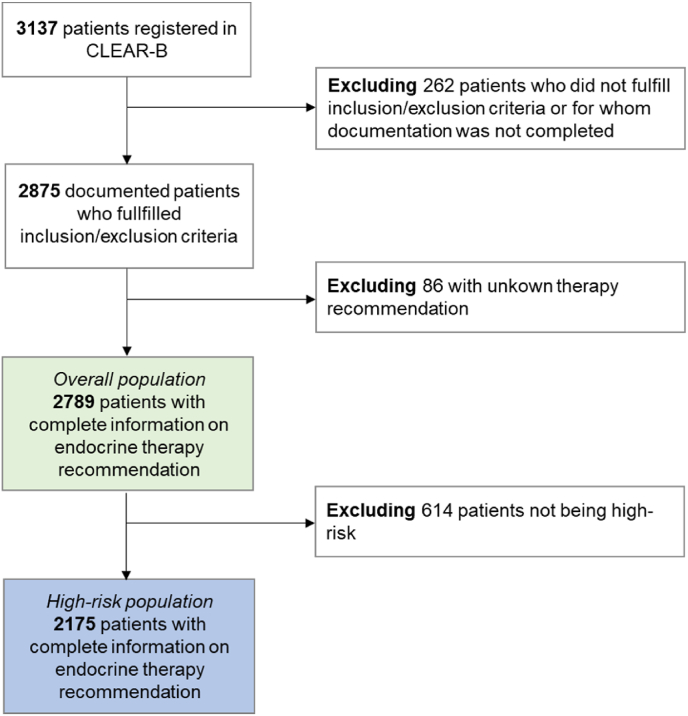
Patient flow chart. A subgroup of patients from the overall population, fulfilling the minimum prognostic criteria to match an indication with a CDK4/6 inhibitor according to the current indication of approved CDK4/6 inhibitors, was assessed in the high-risk subpopulation.

To appraise the choice of endocrine therapy in relation to disease and patient characteristics, all patients with complete documentation and meeting all inclusion and exclusion criteria were evaluated. In addition, a high-risk population, defined according to the minimal criteria for CDK4/6 inhibitor ribociclib therapy ([1] node-positive or node-negative with a tumor ≥5 cm [2], with a tumor of 2–5 cm plus a tumor grading of 3, or [3] with a Ki-67 staining of ≥20 % or a high genomic risk profile according to multigene prognostic testing), was evaluated. Of the 3137 patients enrolled in CLEAR-B, 2789 had complete documentation, of which 2175 were at high risk. The patient flow chart is shown in Fig. 1.

### 2.5 Study endpoints and statistics

The intention-to-treat variables were categorized as TAM monotherapy, TAM + OFS, AI + OFS, or other. The per-treatment variables were categorized as TAM, TAM + OFS, AI + OFS, TAM ± OFS followed by AI + OFS, AI + OFS followed by TAM ± OFS, or other. The patient and disease characteristics were descriptively tabulated with frequencies and percentages. The missing values were also reported in the descriptive analyses. All analyses were performed for the overall population and the high-risk population, and all statistical analyses were carried out using the R system for statistical computing (version 4.3.0, Vienna, 2023).

## 3 Results

### 3.1 Patient characteristics

In the overall population (N = 2789), the patients were 44.5 (±6.1) years old on average. The majority (N = 1493, 55.6 %) had a Ki-67 of ≥20 % and were pretreated with a chemotherapy (N = 1880, 67.5 %). Most patients did not have comorbidities (N = 1673, 61.8 %). Lymph node involvement was documented for 58 % of the patients (N = 1567). None of the patient or tumor characteristics were substantially different between the patients with a primary diagnosis in either 2016–2019 or 2022–2023. All these characteristics are shown in Table 1.

**Table 1:** Patient characteristics of all patients according to the year of primary diagnosis (2016–2019 versus 2022–2023). [BMI: body mass index, SD: standard deviation, ECOG: Eastern Cooperative Oncology Group performance status, N: lymph nodes, T: tumor size, BET: breast conservation therapy, CDK4/6i: CDK4/6 inhibitor].

Characteristic		All patients (N = 2789)	2016–2019 (N = 1717)	2022–2023 (N = 1072)
Age at initial diagnosis (years)	Mean (SD)	44.5 (6.1)	44.6 (6.2)	44.2 (6.0)
BMI (kg/m^2^)	Mean (SD)	25.6 (5.2)	25.5 (5.0)	25.8 (5.5)
ECOG	0	1870 (95.5)	1045 (95.3)	825 (95.6)
	1–4	89 (4.5)	51 (4.7)	38 (4.4)
	Missing	830	621	209
Ki-67	<20 %	1194 (44.4)	708 (43.3)	486 (46.2)
	≥20 %	1493 (55.6)	926 (56.7)	567 (53.8)
	Missing	102	83	19
Tumor grading	G1	342 (12.7)	201 (12.2)	141 (13.5)
	G2	1676 (62.1)	1012 (61.3)	664 (63.5)
	G3	679 (25.2)	439 (26.6)	240 (23.0)
	Missing	92	65	27
Highest T stage	TX	2 (0.1)	2 (0.1)	0 (0.0)
	T0	25 (0.9)	18 (1.1)	7 (0.7)
	Tis	17 (0.6)	14 (0.8)	3 (0.3)
	T1	782 (28.4)	523 (31.0)	259 (24.3)
	T2	1603 (58.2)	956 (56.7)	647 (60.6)
	T3	264 (9.6)	143 (8.5)	121 (11.3)
	T4	60 (2.2)	30 (1.8)	30 (2.8)
	Missing	36	31	5
Highest N status	Nx	3 (0.1)	2 (0.1)	1 (0.1)
	N0	1130 (41.9)	708 (43.2)	422 (39.8)
	N1	1210 (44.8)	713 (43.5)	497 (46.9)
	N > 1	357 (13.2)	217 (13.2)	140 (13.2)
	Missing	89	77	12
Breast cancer anatomic stage	0	16 (0.6)	13 (0.8)	3 (0.3)
	IA	256 (9.2)	177 (10.3)	79 (7.4)
	IB	73 (2.6)	51 (3.0)	22 (2.1)
	IIA without CDK4/6i indication	345 (12.4)	194 (11.3)	151 (14.1)
	IIA with CDK4/6i indication	816 (29.3)	524 (30.5)	292 (27.2)
	IIB	675 (24.2)	387 (22.5)	288 (26.9)
	IIIA	394 (14.1)	227 (13.2)	167 (15.6)
	IIIB	57 (2.0)	28 (1.6)	29 (2.7)
	IIIC	64 (2.3)	36 (2.1)	28 (2.6)
	Not determinable	93 (3.3)	80 (4.7)	13 (1.2)
	Missing	0	0	0
Previous chemotherapy	Yes	1880 (67.5)	1191 (69.4)	689 (64.3)
	No	907 (32.5)	524 (30.6)	383 (35.7)
	Missing	2	2	0
Neoadjuvant chemotherapy	Yes	970 (34.8)	588 (34.2)	382 (35.6)
	No	1819 (65.2)	1129 (65.8)	690 (64.4)
	Missing	0	0	0
Adjuvant chemotherapy	Yes	1014 (36.4)	661 (38.5)	353 (32.9)
	No	1775 (63.6)	1056 (61.5)	719 (67.1)
	Missing	0	0	0
Previous radiotherapy	Yes	2174 (78.2)	1377 (80.5)	797 (74.6)
	No	605 (21.8)	333 (19.5)	272 (25.4)
	Missing	10	7	3
Type of surgery	BET	1691 (61.7)	1099 (65.0)	592 (56.3)
	Mastectomy	976 (35.6)	563 (33.3)	413 (39.3)
	Other or unknown	75 (2.7)	29 (1.8)	47 (4.4)
	Missing	47	26	21
Comorbidities	0	1673 (61.8)	1054 (63.3)	619 (59.4)
	1	642 (23.7)	395 (23.7)	247 (23.7)
	2+	392 (14.5)	216 (12.9)	176 (16.9)
	Missing	82	52	30

Within the high-risk group of patients (N = 2175; Supplementary Table 4), the majority exhibited aggressive disease characteristics. Specifically, 62.0 % (N = 1301) had a Ki-67 index of ≥20 %, indicating high proliferative activity. Moreover, a significant proportion, 74.4 % (N = 1617), had positive lymph node status. Chemotherapy was administered to 76.6 % of the patients (N = 1662). Similar to the overall population, no substantial differences between the patients diagnosed in 2016–2019 and 2022–2023 were found.

### 3.2 Distribution of adjuvant endocrine therapies in 2016–2019 and 2022–2023

The data on the recommended and received endocrine therapies for the overall population are presented in Table 2. Several differences in adjuvant endocrine treatment distributions between the patients diagnosed in 2016–2019 and 2022–2023 were observed. From 2016 to 2019, TAM monotherapy was predominantly recommended (N = 1387, 80.8 %). AI + OFS was recommended for only 8.4 % (N = 145) of the patients, while the combination of TAM + OFS was recommended for 9.3 % (N = 160). The subsequently received therapies were in line with the recommendations, although 244 patients (14.9 %) switched from TAM ± OFS to AI + OFS. In contrast, 42.1 % of the patients diagnosed in 2022–2023 were recommended AI + OFS (N = 451). Additionally, TAM + OFS was recommended more often than in 2016–2019 (N = 177, 16.5 %). Similar to 2016–2019, the received therapies were consistent with the recommended therapies. Notably, even more patients received upfront AI + OFS therapy than were initially recommended (48.6 %, N = 427).

**Table 2:** Endocrine treatments within the overall population according to the year of primary diagnosis (2016–2019 versus 2022–2023). [AI: aromatase inhibitor, TAM: tamoxifen, OFS: ovarian function suppression].

Characteristic		All patients (N = 2789)	2016–2019 (N = 1717)	2022–2023 (N = 1072)
Endocrine therapy as recommended	AI + OFS	596 (21.4)	145 (8.4)	451 (42.1)
	TAM monotherapy	1811 (64.9)	1387 (80.8)	424 (39.6)
	TAM + OFS	337 (12.1)	160 (9.3)	177 (16.5)
	Other	45 (1.6)	25 (1.5)	20 (1.9)
	Missing	0	0	0
Endocrine therapy as received	AI + OFS for 5 years	556 (22.1)	129 (7.9)	427 (48.6)
	TAM monotherapy for 5 years	1666 (66.2)	1236 (75.5)	430 (49.0)
	AI + OFS followed by TAM ± OFS	21 (0.8)	16 (1.0)	5 (0.6)
	TAM ± OFS followed by AI + OFS	256 (10.2)	244 (14.9)	12 (1.4)
	Other	16 (0.6)	12 (0.7)	4 (0.5)
	Missing	274	80	194

In the high-risk subpopulation (Supplementary Table 5), the therapy distributions of the patients diagnosed in 2016–2019 were similar to that of the overall population. Therapy with AI + OFS was recommended for 9.4 % (N = 119) and TAM monotherapy for 79.1 % (N = 1002). For the high-risk patients diagnosed in 2022–2023, AI + OFS therapy was most commonly recommended (N = 410, 49.0 %). During this period, the majority of patients (N = 389; 55.7 %) received upfront AI + OFS therapy.

### 3.3 Therapy recommendations according to patient and tumor characteristics

The recommended therapies in relation to patient and tumor characteristics for the overall population are shown in Table 3. Age differed between the treatment recommendations. The patients who received recommendations for TAM + OFS were the youngest (40.3 ± 6.4 years old), while those who were recommended TAM monotherapy or AI + OFS combination therapy were of similar age (45.4 ± 5.5 years and 43.9 ± 6.6 years old, respectively). Recommendations for AI + OFS were commonly given to node-positive patients (N = 425, 72.3 %), although node positivity was also common in the TAM monotherapy (52.3 %, N = 910) and TAM + OFS (62.0 %, N = 204) recommendations. Regarding disease stage, the majority of patients recommended to be treated with AI + OFS were stage IIB or higher (N = 375, 62.9 %), while only 35.6 % (N = 645) with stage IIB or higher received TAM monotherapy recommendations. For the patients who received TAM + OFS recommendations, 43.9 % (N = 148) were classified as high risk (stage IIB or higher).

**Table 3:** Patient characteristics of the overall population according to the recommended therapy. Patients for whom “other endocrine therapy” was recommended (N = 45) were omitted from the analyses*.* [BMI: body mass index, SD: standard deviation, ECOG: Eastern Cooperative Oncology Group performance status, N: lymph nodes, T: tumor size, BET: breast conservation therapy, CDK4/6i: CDK4/6 inhibitor, AI: aromatase inhibitor, TAM: tamoxifen, OFS: ovarian function suppression].

Characteristic		AI + OFS (N = 596)	TAM monotherapy (N = 1811)	TAM + OFS (N = 337)
Age at initial diagnosis (years)	Mean (SD)	43.9 (6.6)	45.4 (5.5)	40.3 (6.4)
BMI (kg/m^2^)	Mean (SD)	25.9 (5.5)	25.6 (5.1)	25.2 (5.2)
ECOG	0	434 (94.8)	1153 (95.8)	249 (95.0)
	1–4	23 (5.2)	50 (4.2)	13 (5.0)
	Missing	138	608	75
Ki-67	<20 %	212 (36.2)	830 (48.0)	138 (41.4)
	≥20 %	373 (63.8)	900 (52.0)	195 (58.6)
	Missing	11	81	4
Tumor grading	G1	42 (7.2)	255 (14.6)	38 (11.5)
	G2	373 (64.0)	1087 (62.4)	195 (58.9)
	G3	168 (28.8)	400 (23.0)	98 (29.6)
	Missing	13	69	6
Highest T	TX	0 (0.0)	2 (0.1)	0 (0.0)
	T0	8 (1.4)	12 (0.7)	5 (1.5)
	Tis	0 (0.0)	15 (0.8)	2 (0.6)
	T1	122 (20.6)	545 (30.5)	103 (31.0)
	T2	348 (58.8)	1038 (58.2)	189 (56.9)
	T3	87 (14.7)	144 (8.1)	29 (8.7)
	T4	27 (4.6)	29 (1.6)	4 (1.2)
	Missing	4	26	5
Highest N status	Nx	0 (0.0)	3 (0.2)	0 (0.0)
	N0	163 (27.7)	827 (47.5)	125 (38.0)
	N1	284 (48.3)	736 (42.3)	167 (50.8)
	N > 1	141 (24.0)	174 (10.0)	37 (11.2)
	Missing	8	71	8
Breast cancer anatomic stage	0	3 (0.5)	10 (0.6)	3 (0.9)
	IA	28 (4.7)	185 (10.2)	38 (11.3)
	IB	10 (1.7)	52 (2.9)	11 (3.3)
	IIA without CDK4/6i indication	37 (6.2)	281 (15.5)	24 (7.2)
	IIA with CDK4/6i indication	135 (22.7)	563 (31.1)	105 (31.2)
	IIB	176 (29.5)	393 (21.7)	93 (27.6)
	IIIA	148 (24.8)	189 (10.4)	48 (14.2)
	IIIB	26 (4.4)	27 (1.5)	4 (1.2)
	IIIC	25 (4.2)	36 (2.0)	3 (0.9)
	Not determinable	8 (1.3)	75 (4.1)	8 (2.4)
	Missing	0	0	0
Previous chemotherapy	Yes	446 (75.0)	1141 (63.0)	256 (76.0)
	No	149 (25.0)	669 (37.0)	81 (24.0)
	Missing	1	1	0
Neoadjuvant chemotherapy	Yes	288 (48.3)	514 (28.4)	149 (44.2)
	No	308 (51.7)	1297 (71.6)	188 (55.8)
	Missing	0	0	0
Adjuvant chemotherapy	Yes	199 (33.4)	675 (37.3)	119 (35.3)
	No	397 (66.6)	1136 (62.7)	218 (64.7)
	Missing	0	0	0
Previous radiotherapy	Yes	465 (78.4)	1411 (78.2)	261 (77.7)
	No	128 (21.6)	394 (21.8)	75 (22.3)
	Missing	3	6	1
Type of surgery	BET	307 (52.7)	1164 (65.3)	191 (57.2)
	Mastectomy	258 (44.3)	570 (32.0)	136 (40.7)
	Other	18 (3.1)	45 (2.5)	6 (1.8)
	Unknown	0 (0.0)	3 (0.2)	1 (0.3)
	Missing	13	29	3
Comorbidities	0	327 (56.7)	1108 (63.0)	215 (65.3)
	1	150 (26.0)	409 (23.3)	74 (22.5)
	2+	100 (17.4)	241 (13.7)	40 (12.2)
	Missing	19	53	8

The respective data for the high-risk subpopulation are shown in Supplementary Table 6.

### 3.4 Dynamics of therapy recommendations over time

Detailed information about patient and tumor characteristics in relation to therapy recommendations for all the patients diagnosed in 2016-2019 and 2022–2023 are presented in Supplementary Table 7 and Supplementary Table 8, respectively.

**Fig. 2:**
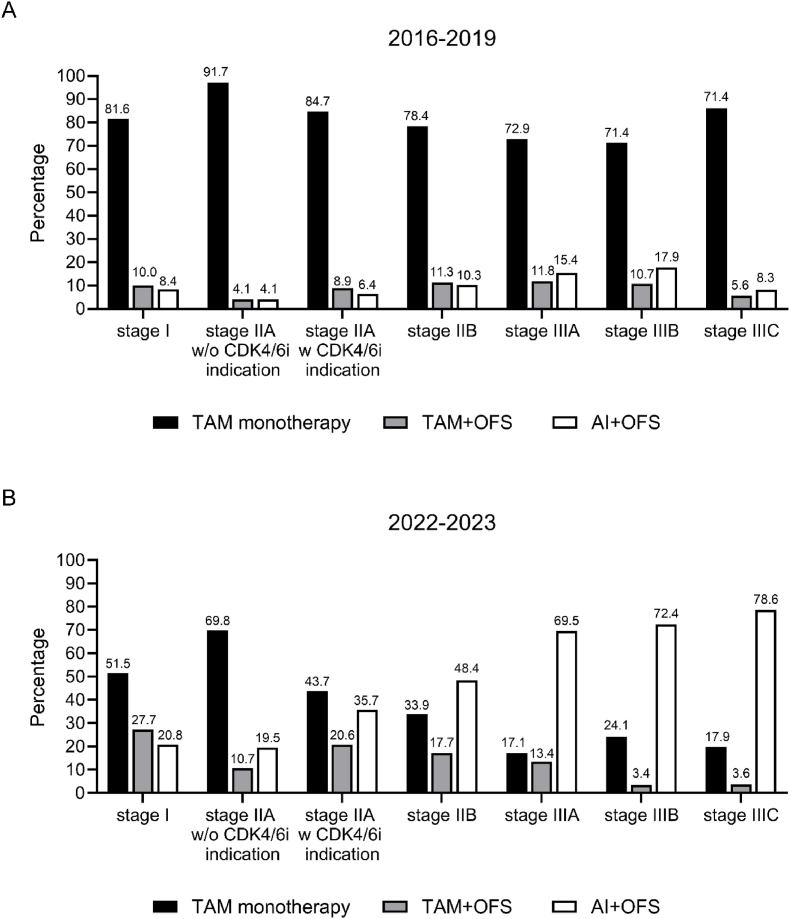
Distribution of therapy recommendations in the overall population according to tumor stage in the years (A) 2016–2019 and (B) 2022–2023 (figures based on the case numbers of Supplementary Tables 7 and 8).

From 2016 to 2019 to 2022–2023, the use of AI + OFS in all tumor-stage subgroups increased (Fig. 2). In 2016–2019, no clear correlation between tumor stage and the distribution of the recommended adjuvant endocrine therapies was observed. Even among the high-risk patients, such as stage III, AI + OFS was only recommended for 14.7 % (Fig. 2A). Interestingly, a correlation between tumor stage and recommended therapies was observed for the patients diagnosed in 2022–2023. While 20–21 % of the patients with stage I or low-risk stage IIA received an AI + OFS recommendation (Fig. 2B), such a recommendation increased to 35.7 % for high-risk stage IIA and to 48.4 % for stage IIB. Overall, the majority of patients with higher tumor stages received AI + OFS recommendations (69.5 %, 72.4 %, and 78.6 % for stages IIIA, IIIB, and IIIC, respectively).

## 4 Discussion

In this retrospective analysis of premenopausal patients with HRpos/HER2neg eBC and an increased risk of recurrence, we demonstrated that AI + OFS is currently the recommended standard adjuvant endocrine treatment. Conversely, TAM monotherapy was previously (2016–2019) the most frequently recommened therapy, which is in contrast to the guidelines. The frequency of the choice of recommended therapy did not depend on the risk of recurrence from 2016 to 2019.

In the overall population, AI + OFS usage saw a remarkable increase from 8.4 % in 2016-2019 to 42.1 % in 2022–2023, while TAM monotherapy usage decreased from 80.8 % to 39.6 %. Additionally, TAM + OFS usage increased from 9.3 % to 16.5 %. Even more interesting, in the high-risk subset, AI + OFS usage increased significantly from 9.4 % to 49.0 %, while TAM monotherapy usage decreased from 79.1 % to 32.4 %. This shift indicates a growing preference for more advanced and more effective endocrine therapies over TAM as a monotherapy.

The low recommendation rate of AI + OFS for patients diagnosed in 2016–2019 in our study confirms previously published data on the high-risk population of the monarchE study in which approximately 22 % of premenopausal patients were treated with AI + OFS [25]. In our study, the recommendation rate for AI + OFS for stage III patients in 2016–2019 (most similar to the monarchE population) was approximately 15 %. Notably, our data represent therapy recommendations in clinical routines that are closer to the German real-world setting than the monarchE study data. Data from 2017 to 2019 in Paluch-Shimon et al.‘s study showed that Germany was not the only country with a low utilization rate of AI + OFS. France and Turkey had similarly low rates, and in Denmark, the utilization rate was as low as 2 % [25]. In contrast, the rate of AI + OFS usage was almost 90 % in Italy and approximately 55 % in the United States. It can only be assumed that in Germany, the available evidence was not considered strong enough to warrant AI + OFS in this higher risk population.

Substantial evidence shows that AI + OFS is one of the most effective therapies for patients with HRpos/HER2neg eBC (SOFT and TEXT [26,27]). The most recent update indicated improved DFS and distant recurrence-free interval for AI + OFS over TAM + OFS. Although OS was comparable in the overall population, some high-risk patient groups showed a benefit with AI + OFS compared to TAM + OFS (i.e., women younger than 35 years (4.0 %) and those with >2 cm (4.5 %) or grade 3 tumors (5.5 %)) [28]. Interestingly, the benefit was particularly pronounced in patients who had received neoadjuvant chemotherapy [29]. In addition, the ABCSG-12 and HOBOE studies contribute to the existing body of knowledge regarding the most efficacious endocrine therapy for premenopausal patients with eBC [30,31]. The Early Breast Cancer Trialists Collaborative Group recently analyzed data pertaining to more than 7000 patients and evaluated whether AI or TAM was more favorable as an adjuvant treatment. This analysis had a median follow-up time of 8.0 years and showed that the patients' 10-year recurrence rate was 14.7 % for AI + OFS versus 17.5 % for TAM + OFS [21]. Furthermore, the relative risk (RR) for recurrence was 0.79 (95 %CI: 0.69–0.90) in favor of AI + OFS, and this group had less distant recurrences (RR = 0.83; 95 %CI: 0.69–0.90). However, overall survival was not significantly different between AI + OFS and TAM + OFS, as the 10-year death rate with AI + OFS was 6.8 % compared to 7.2 % with TAM + OFS [21]. The patients treated with AI + OFS exhibited a higher incidence of bone fractures and osteoporosis, while those treated with tamoxifen demonstrating a greater prevalence of endometrial abnormalities [21]. Notably, monitoring all premenopausal patients’ quality of life is crucial, as long-term quality of life appears to be primarily influenced by adjuvant endocrine treatment rather than previous adjuvant chemotherapy [32].

The data from these studies have been incorporated into most national and international guideline recommendations [[33], [34], [35], [36], [37]] and the level of evidence for these recommendations evolved with updates to the data over the years. However, while most guidelines refer to patients treated with TAM + OFS or AI + OFS as “patients with an increased risk,” the precise definition of this term is unclear. The perception of risk can vary significantly between different physicians. The introduction of novel therapies in the adjuvant setting, such as abemaciclib and ribociclib, highlights the importance of patient prognostication when discussing these therapies. Both substances have demonstrated substantial benefits for the respective patient populations [23,[38], [39], [40]], suggesting that the risk of recurrence risk may need to be considered in the choice of adjuvant endocrine treatment. The pivotal CDK4/6 inhibitor trials—monarchE for abemaciclib and NATALEE for ribociclib—focused on populations with a high risk of recurrence. A recent analysis of a large cohort of postmenopausal patients with HRpos/HER2neg eBC and receiving upfront AI—considered a higher-risk population—found that 13 % would have been eligible for monarchE and 32 % for NATALEE [41]. Thus, these populations can be considered as patients with an increased recurrence risk. In light of this, the use of TAM as an endocrine combination therapy in monarchE may be regarded as somewhat controversial.

Our study has strengths and weaknesses. A sample size of >3000 patients from >55 study sites across Germany is sufficient for making robust statements. Indeed, the inclusion criteria were designed to select all patients with an increased recurrence risk, thereby ensuring that this population is representative. Despite the study's retrospective nature being a limitation, the selection process ensured that all participating study sites had a prospective overview of all treated patients with the respective patient characteristics, reducing the risk of bias in this aspect. In addition, the vast majority of breast cancer patients in Germany are treated in certified breast centers, further reducing the risk of selection bias. Nevertheless, as this analysis does not provide an assessment of prognosis under the chosen therapies, it cannot contribute to the discussion of which therapy is more effective. Therefore, future analyses will be important to generate more data on the efficacy of the described therapy options.

In conclusion, the CLEAR-B study sheds light on a paradigm shift in Germany's treatment approach for HRpos/HER2neg eBC patients with a higher recurrence risk. While TAM monotherapy was the predominant treatment in 2016–2019, real-world data from 2022 to 2023 from the same treatment centers indicates that AI + OFS is now the standard treatment for the majority of patients with an increased and high recurrence risk.

## CRediT authorship contribution statement

**Volkmar Müller:** Writing – review & editing, Investigation, Funding acquisition, Conceptualization. **Manuel Hörner:** Writing – review & editing, Project administration, Investigation, Conceptualization. **Marc Thill:** Writing – review & editing, Investigation. **Maggie Banys-Paluchowski:** Writing – review & editing, Investigation. **Sabine Schmatloch:** Writing – review & editing, Investigation. **Peter A. Fasching:** Writing – review & editing, Writing – original draft, Project administration, Investigation, Funding acquisition, Conceptualization. **Nadia Harbeck:** Writing – review & editing, Investigation. **Dagmar Langanke:** Writing – review & editing, Investigation. **Sabrina Uhrig:** Writing – review & editing, Data curation. **Lothar Häberle:** Writing – review & editing, Formal analysis. **Dorothea Fischer:** Writing – review & editing, Investigation. **Alexander Hein:** Writing – review & editing, Investigation. **Tanja N. Fehm:** Writing – review & editing, Investigation. **Chloë Goossens:** Writing – review & editing, Writing – original draft, Investigation. **Jürgen Terhaag:** Writing – review & editing, Investigation. **Uwe Heilenkötter:** Writing – review & editing, Investigation. **Peter Dall:** Writing – review & editing, Investigation. **Christian Rudlowski:** Writing – review & editing, Investigation. **Rachel Wuerstlein:** Writing – review & editing, Investigation. **Mustafa Aydogdu:** Writing – review & editing, Investigation. **Mignon-Denise Keyver-Paik:** Writing – review & editing, Investigation. **Carolin Hammerle:** Writing – review & editing, Investigation. **Natalija Deuerling:** Writing – review & editing, Investigation. **Elmar Stickeler:** Writing – review & editing, Investigation. **Bahriye Aktas:** Writing – review & editing, Investigation. **Erik Belleville:** Writing – review & editing, Writing – original draft, Project administration. **Martin Thoma:** Writing – review & editing, Investigation. **Nina Ditsch:** Writing – review & editing, Investigation. **Yasmin Baila:** Writing – review & editing, Investigation. **Christian Roos:** Resources, Project administration. **Christian Mann:** Resources, Project administration. **Caterina Iuliano:** Resources, Project administration. **Sara Y. Brucker:** Writing – review & editing, Investigation. **Andreas Schneeweiss:** Writing – review & editing, Investigation. **Andreas D. Hartkopf:** Writing – review & editing, Investigation, Conceptualization.

## Declaration of competing interest

**V.M.** received personal fees from Novartis, during the conduct of the study; received speaker honoraria from Amgen, AstraZeneca, Daiichi Sankyo, Eisai, Pfizer, MSD, Novartis, Roche, Teva, Seagen, GSK, Gilead; received consultancy honoraria from Genomic Health, Gilead, Hexal, Roche, Pierre Fabre, Amgen, ClinSol, Novartis, MSD, Daiichi-Sankyo, Eisai, Lilly, GSK, Gilead; received institutional research support fromNovartis, Roche, Seagen, Genentech, outside the submitted work. **M.H.** received travel support from Novartis, Lilly Deutschland GmbH, and AstraZeneca. **M.T.** participated on advisory boards for Agendia, AstraZeneca, Clovis, Daiichi Sanyo, Eisai, Gilead Science, GSK, Lilly, MSD, Novartis, Organon, Pfizer, Pierre Fabre, Seagen, and Roche and received honoraria for lectures from Agendia, Amgen, Clovis, Daiichi Sankyo, Eisai, GSK, Hexal, Lilly, MSD, Roche, Novartis, Organon, Pfizer, Seagen, Exact Sciences, Viatris, Vifor, and AstraZeneca; trial funding from Exact Sciences and Endomag; and manuscript support from Amgen, ClearCut, pfm medical, Roche, Servier, and Vifor. **M. B.-P.** received research support from Damp Stiftung, Claudia von Schilling Foundation for Breast Cancer Research, Ehmann-Stiftung Savognin, EndoMag, Mammotome, MeritMedical, Sirius Medical, Gilead, Hologic, ExactSciences; received consulting fees from Roche, Novartis, Pfizer, pfm, Eli Lilly, Onkowissen, Seagen, AstraZeneca, Eisai, Amgen, Samsung, Canon, MSD, GSK, Daiichi Sankyo, Gilead, Sirius Medical, Syantra, resitu, Pierre Fabre, ExactSciences, Menarini Stemline, if-kongress, Jörg Eickeler; received speaker honoraria from: Roche, Novartis, Pfizer, pfm, Eli Lilly, Onkowissen, Seagen, AstraZeneca, Eisai, Amgen, Samsung, Canon, MSD, GSK, Daiichi Sankyo, Gilead, Sirius Medical, Syantra, resitu, Pierre Fabre, ExactSciences, Menarini Stemline, if-kongress, Jörg Eickeler; received travel support from: Eli Lilly, ExactSciences, Pierre Fabre, Pfizer, Daiichi Sankyo, Roche; participated in advisory boards for Roche, Novartis, Pfizer, pfm, Eli Lilly, Onkowissen, Seagen, AstraZeneca, Eisai, Amgen, Samsung, Canon, MSD, GSK, Daiichi Sankyo, Gilead, Sirius Medical, Syantra, resitu, Pierre Fabre, ExactSciences, Menarini Stemline; has a role in AGO Breast Committee, S3 guideline expert panel, Board of Directors and 1. Vice-Chair of the AWOgyn, Vice-Chair of EUBREAST e.V. Study Group. **P.A.F.** received personal fees from Novartis, Pfizer, Daiichi Sankyo, AstraZeneca, Eisai, Merck Sharp & Dohme, Lilly, Pierre Fabre, Seagen, Roche, Hexal, Agendia, and Gilead and grants from Biontech and Cepheid. **N.H.** received honoraria for lectures and/or consulting from AstraZeneca, Daiichi Sankyo, Gilead, Lilly, MSD, Novartis, Pierre Fabre, Pfizer, Roche, Seagen, Viatris, and Zuelligpharma and is a co-director of Westdeutsche Studiengruppe. **D.L.** received honoraria for lectures/presentations from Lilly Deutschland GmbH, Pfizer Pharma GmbH, Roche Pharma GmbH, Daiichi Sankyo GmbH, AstraZeneca GmbH and Gilead Science. Travel support from Gilead Science and Pfizer Pharma GmbH. Participated in advisory boards for Roche Pharma GmbH, Lilly Deutschland GmbH and Gilead Science **L.H.** is a shareholder of Biomed Statistics GmbH. **T.N.F.** received honoraria for lectures from Onkowissen, Medconcept, FOMF. She redeived travel support by Roche, Daichii Sankyo.participated on advisory boards for Amgen, Daiichi Sankyo, Novartis, Pfizer, and Roche and received honoraria for lectures from Amgen, Celgene, Daiichi Sankyo, Roche, Novartis, and Pfizer. **C.G.** received honoraria for lectures from Novartis Pharma and ClinSol GmbH & Co. KG. **P.D.** received honoraria from MSD, Pierre Fabre, Novartis, AstraZeneca, Lilly, Gilead, Pfizer, and Roche. **C.Ru.** received research support from Novartis; received honorario from Lilly, Novartis AG; received travel support from Lilly; participated in advisory boards for Bayer AG. **R.W.** received consulting fees/travel support from Agendia, Amgen, AstraZeneca, Daiichi Sankyo, Exact Sciences, Hexal, Lilly, MSD, Nanostring, Novartis, Paxman, Pfizer, Riemser, Roche, Sidekick, Teva, Viatris; received speaker/lecture honoraria from ClinSol GmbH & Co. KG, Onkowissen, H + O Communications, Streamed up; has a role in AGO, WSG, PINK, Brustrkebs Deutschland, Stiftung Junge Erwachsene mit Krebs, AGSMO.**E.S.** honoraria from Roche, Celgene, AstraZeneca, Novartis, Pfizer, Tesaro, Aurikamed GmbH, Pfizer, Seagen, Pierre Fabre, MCI Deutschland GmbH, bsh medical communications GmbH, Onkowissen TV. **B.A.** received honoraria and travel grants for clinical research management and/or medical education activities from Amgen, AstraZeneca, Daiichi Sankyo, Eisai, Genomic Health, Gilead, GSK, Lilly, Medtronic, MSD, Novartis, Onkowissen, Pfizer, Roche, Seagen, Stemline, and Tesaro. **E.B.** received honoraria from Gilead, Ipsen, Sanofi, Sandoz, SunPharma, AstraZeneca, Novartis, Hexal, BMS, Lilly, Pfizer, Roche, MSD, BBraun, and onkowissen.de. **M.T.** received research support from Exanct Sciences, Endomag; consulting fees from Agendia, Amgen, AstraZeneca, Becton and Dickinson, Clearcut,Daiichi Sankyo, Eisai, Endomag, Exact Sciences, Gilead Sciences, Grünenthal, GSK, Lilly, Norgine, Neodynamics, Novartis, Onkowissen, Organon, Pfizer, Pfm medical, Pierre Fabre, Roche, RTI Surgical, Seagen, Sirius Pintuition, Sysmex; speaker/presentation honoraria from Amgen, AstraZeneca, Connect Medica, Eisai, Exact Sciences, Endomag, Gedeon Richter, Gilead Sciences, GSK, Hexal, I-Med-Institute, Joerg Eickeler, Lilly, MCI, Medscape, MDS,Medtronic, Novartis, Onkowissen, Pfizer, Pfm medical, Roche, Seagen, Streamed UP, Sirius Medical, Sysmex, Vifor, Viatris, Servier; received payment for expert testimony from RTI Surgival, Pfm medical; received travel support from: Amgen, AstraZeneca, Celgene, Daiichi Sankyo, Hexal, Neodynamis, Clearcut, Novartis, Pfizer, Roche, Eisai, Exact Sciences, Art Tempi, Pfm medical, Roche, Hexal, MCI, Lilly, MSD, Norgine, Novartis, Pfizer, RTI Surgical, Seagen, Sirius Medical; has a role in OncoNet Rhein Main, AWOgyn, DGGG, BLFG. **N.D.** received consulting fees from Roche, pmf medical, ClinSol GmbH & Co. KG, Onkowissen; received speaker/lecture honoraria from MSD, Medi-Seminar, Merit-Medical, Pfizer, Seagen, Gilead, Roche, Novartis, Pierre-Fabre, Exact Sciences, AstraZeneca, Eickeler-Kongress, Lilly, Novartis; received travel support from Roche, Pfizer, Gilead, Lilly, Novartis, AstraZeneca, Menarini-Stemline; participated on advisory boards for Novartis; has a role in EUBREAST, Frauenselbsthilfe gegen Krebs, Brustkrebs Deutschland and Brustkrebs München e.V. **A.S.** received research grants from Celgene and Roche; honoraria from Amgen, AstraZeneca, Aurikamed, Bayer, Celgene, ClinSol, Clovis Oncology, coma UroGyn, Connectmedica, Daiichi Sankyo, Gilead, GSK, if-kongress, I-MED, iOMEDICO, Lilly, MCI Deutschland, med publico, Metaplan, MSD, Mylan, NanoString Technologies, Novartis, onkowissen.de, Pfizer, Pierre Fabre, promedicis, Roche, Seagen, streamedup, Tesaro; and travel support from AstraZeneca, Celgene, Daiichi Sankyo, Gilead, Pfizer, Roche **A.D.H.** received speaker and consultancy honoraria from AstraZeneca, Genomic Health, Roche, Novartis, Celgene, Lilly, MSD, Eisai, Teva, Tesaro, Daiichi-Sankyo, Hexal and Pfizer. The remaining authors have nothing to disclose.
