# Supplementary material for: Real-world utilization of aromatase inhibitors, tamoxifen, and ovarian function suppression in premenopausal patients with early hormone receptor-positive, HER2-negative breast cancer with increased recurrence risk
Source: Breast. 2025 Mar 22;81:104458. doi: 10.1016/j.breast.2025.104458 (PMC11986623; doi:10.1016/j.breast.2025.104458)
Supplement: Multimedia component 1 [file mmc1.docx]

# **Supplementary Material**

**Supplementary Table 1**: Participating study sites of the CLEAR-B study in alphabetical order.

| Aachen, Universitätsklinikum Aachen, Klinik für Gynäkologie und Geburtsmedizin | Hanau, Klinikum, Gynäkologie |
| --- | --- |
| Ansbach, ANregiomed, Onkologisches Zentrum | Heidelberg, Universitätsklinik, Frauenklinik/NCT |
| Aschaffenburg, Klinikum, Frauenklinik | Heppenheim, Kreiskrankenhaus Bergstraße GmbH, Gynäkologie |
| Augsburg, Universitätsklinik, Frauenklinik | Hilden, St. Josefs Krankenhaus, Brustzentrum |
| Bad Homburg, Hochtaununs Kliniken, Gynäkologie | Itzehoe, Holsteinisches Brustzentrum |
| Bergisch Gladbach, Evangelisches Krankenhaus, Gynäkologie | Kassel, Elisabeth-Krankenhaus, Frauenheilkunde |
| Biberach, Med. Kompetenz Zentrum, Hämatologie | Kassel, Gesundheit Nordhessen, Frauenheilkunde und Geburtshilfe |
| Bottrop, Marienhospital, Brust und Gynäkologisches Krebszentrum | Krefeld, Helios Klinikum Krefeld, Gynäkologisches Krebszentrum |
| Bremen, DIAKO, Brustzentrum | Landshut, Krankenhaus, VK&K Studien GbR |
| Bremen, Klinikum Bremen-Mitte, Brustzentrum | Leipzig, St. Elisabeth Krankenhaus Leipzig, Brustzentrum |
| Bruchsal, RKH Gesundheit, Frauenheilkunde | Leipzig, Universitätsklinik, Frauenheilkunde |
| Buchholz, Krankenhaus Buchholz, Frauenheilkunde | Lingen, Bonifatiushospital, Frauenheilkunde |
| Celle, AKH Klinik, Brustzentrum | Lübeck, Universitätsklinikum Schleswig-Holstein, Frauenklinik |
| Chemnitz, Klinikum Chemnitz, Frauenheilkunde und Geburtshilfe | Lüneburg, Klinikum, Brustzentrum |
| Cottbus, Carl-Thiem-Klinikum, Gynäkoonkologie | Marktredwitz, Klinikum Fichtelgebirge, Brustzentrum |
| Dresden, Diakonissenkrankenhaus, Gynäkologisches Krebszentrum | München, Universität, Klinik und Poliklinik für Frauenheilkunde |
| Düsseldorf, Universitätsklinik, Frauenheilkunde und Geburtshilfe | Nürnberg, Klinikum Nürnberg, Brustzentrum |
| Ebersbach/Ostsachsen, Klinik, Brustzentrum | Potsdam, Klinikum Ernst von Bergmann, Frauenheilkunde |
| Ebersberg, Kreisklinik, Brustzentrum | Regensburg, Caritas-Krankenhaus St. Josef |
| Eggenfelden, MVZ Eggenfelden | Rostock, Klinikum Südstadt, Brustzentrum |
| Ehingen, Alb-Donau Klinikum, Brustzentrum | Salzwedel, Altmarkt-Klinikum, Brustzentrum |
| Erlangen, Universitätsklinik, Frauenklinik | Traunstein, Kliniken Süd-Ost-Bayern, Frauenklinik |
| Esslingen, Klinikum, Frauenheilkunde und Geburtshilfe | Trier, Klinikum Mutterhaus der Borromäerinnen, Brustzentrum |
| Frankfurt, Agaplesion Diakonie Kliniken gemeinützige GmbH | Tübingen, Universitätsklinik, Frauenklinik |
| Freudenstadt, Klinikum, Gynäkologische Onkologie | Ulm, Universitätsklinik, Frauenheilkunde und Geburtshilfe |
| Halle, Universitäsklinikum Halle, Universitätsklinik und Poliklinik für Gynäkologie | Westernede, Ammerland-Klinik, Frauenklinik |
| Hamburg, Asklepios Klinik Barmbek | Wiesbaden, St. Josefs-Hospital, Brustzentrum |
| Hamburg-Eppendorf, Universitätsklinikum, Gynäkologie | Wolfsburg, Klinikum, MVZ WOB GmbH |

**Supplementary Table 2**: Complete inclusion and exclusion criteria of the CLEAR-B study.

| **Patient inclusion criteria (all had to be fulfilled)** | |
| --- | --- |
|  | Female patient with a first primary diagnosis of hormone receptor-positive, HER2-negative unilateral early breast cancer for whom an endocrine treatment is indicated |
|  | Patient must be at least 18 years of age but not older than 60 |
|  | Premenopausal defined as (**all statements must be true**):   - No oophorectomy before the diagnosis of breast cancer - Women with uterus: regular, physiological menses at the timepoint of primary diagnosis (in case of chemotherapy-induced amenorrhea at the timepoint of the endocrine treatment start, premenopausal status needs to have been affirmed by clinical assessment or ensured by endocrine laboratory confirmation) - Women without uterus and remaining ovaries: premenopausal hormone levels must have been measured at the time of therapy decision for anti-endocrine treatment |
|  | Patient with intermediate- or high-risk early-stage breast cancer defined as (at least one of the following must be fulfilled):   - (Neo)adjuvant chemotherapy - pT≥2cm at the timepoint of definitive surgery - At least one positive lymph node at the time of definitive surgery (pN+) |
|  | Patient must be previously registered in and must have been documented as part of the certification process for a certified breast cancer center according to Deutsche Krebsgesellschaft/Deutsche Gesellschaft für Senologie |
|  | Breast cancer must have been diagnosed either from January 2016 to June 2019 **or** from January 2022 to December 2023 |
| **Exclusion criteria** | |
|  | Patient with a low recurrence risk (see inclusion criteria for definition) |
|  | Locally advanced breast cancer or distant metastases at diagnosis |
|  | Male biological sex |
|  | Patient not treated in a certified breast cancer center |
|  | Previous diagnosis of invasive breast cancer or in situ breast cancer |
|  | Concurrent invasive malignancy |
|  | Bilateral breast cancer at the timepoint of diagnosis |
|  | Previous diagnosis of invasive breast cancer or in situ breast cancer is not allowed |

**Supplementary Table 3:** Requested documentation categories in the CLEAR-B study.

| **Documentation categories** |
| --- |
| 1. Date period of diagnosis |
| 1. Breast cancer stage |
| 1. Tumor biology |
| 1. Neo(adjuvant) chemotherapy |
| 1. Type of surgery |
| 1. Radiation therapy |
| 1. Medical history, including comorbidities and co-medication |
| 1. Start and end of endocrine therapy (to be ascertained and/or confirmed at the timepoint of documentation) |
| 1. Recurrence status at the timepoint of documentation |
| 1. Survival status at the timepoint of documentation |

**Supplementary Table 4:** Patient characteristics of the high-risk subset of patients according to the year of primary diagnosis (2016–2019 versus 2022–2023) [BMI: body mass index, SD: standard deviation, ECOG: Eastern Cooperative Oncology Group performance status, N: lymph nodes, T: tumor size, BET: breast conservation therapy]

| **Patient characteristic** |  | **All patients (N=2175)** | **2016–2019 (N=1303)** | **2022–2023 (N=872)** |
| --- | --- | --- | --- | --- |
| Age at initial diagnosis (years) | Mean (SD) | 44.3 (6.1) | 44.4 (6.2) | 44.1 (6.0) |
| BMI (kg/m^2^) | Mean (SD) | 25.7 (5.2) | 25.5 (5.0) | 25.9 (5.5) |
| ECOG | 0 | 1457 (95.5) | 767 (95.5) | 690 (95.4) |
|  | 1–4 | 69 (4.5) | 36 (4.5) | 33 (4.6) |
|  | Missing | 649 | 500 | 149 |
| Ki-67 | <20% | 798 (38.0) | 461 (37.1) | 337 (39.4) |
|  | ≥20% | 1301 (62.0) | 782 (62.9) | 519 (60.6) |
|  | Missing | 76 | 60 | 16 |
| Tumor grading | G1 | 190 (8.9) | 113 (8.9) | 77 (9.0) |
|  | G2 | 1354 (63.7) | 795 (62.7) | 559 (65.0) |
|  | G3 | 583 (27.4) | 359 (28.3) | 224 (26.0) |
|  | Missing | 48 | 36 | 12 |
| Highest T | TX | 0 (0.0) | 0 (0.0) | 0 (0.0) |
|  | T0 | 17 (0.8) | 12 (0.9) | 5 (0.6) |
|  | Tis | 8 (0.4) | 5 (0.4) | 3 (0.3) |
|  | T1 | 485 (22.3) | 317 (24.3) | 168 (19.3) |
|  | T2 | 1316 (60.5) | 780 (59.9) | 536 (61.5) |
|  | T3 | 286 (13.1) | 158 (12.1) | 128 (14.7) |
|  | T4 | 63 (2.9) | 31 (2.4) | 32 (3.7) |
|  | Missing | 0 | 0 | 0 |
| Highest N status | N0 | 558 (25.7) | 351 (26.9) | 207 (23.7) |
|  | N1 | 1224 (56.3) | 714 (54.8) | 510 (58.5) |
|  | N>1 | 393 (18.1) | 238 (18.3) | 155 (17.8) |
|  | Missing | 0 | 0 | 0 |
| Breast cancer anatomic stage | IIA | 882 (40.6) | 563 (43.2) | 319 (36.6) |
|  | IIB | 733 (33.7) | 422 (32.4) | 311 (35.7) |
|  | IIIA | 426 (19.6) | 248 (19.0) | 178 (20.4) |
|  | IIIB | 60 (2.8) | 30 (2.3) | 30 (3.4) |
|  | IIIC | 74 (3.4) | 40 (3.1) | 34 (3.9) |
|  | Missing | 0 | 0 | 0 |
| Previous chemotherapy | Yes | 1662 (76.6) | 1023 (78.8) | 639 (73.4) |
|  | No | 507 (23.4) | 275 (21.2) | 232 (26.6) |
|  | Missing | 6 | 5 | 1 |
| Neoadjuvant chemotherapy | Yes | 856 (39.4) | 499 (38.3) | 357 (40.9) |
|  | No | 1319 (60.6) | 804 (61.7) | 515 (59.1) |
|  | Missing | 0 | 0 | 0 |
| Adjuvant chemotherapy | Yes | 911 (41.9) | 582 (44.7) | 329 (37.7) |
|  | No | 1264 (58.1) | 721 (55.3) | 543 (62.3) |
|  | Missing | 0 | 0 | 0 |
| Previous radiotherapy | Yes | 1726 (80.1) | 1054 (81.8) | 672 (77.6) |
|  | No | 429 (19.9) | 235 (18.2) | 194 (22.4) |
|  | Missing | 20 | 14 | 6 |
| Type of surgery | BET | 1238 (57.7) | 771 (59.8) | 467 (54.4) |
|  | Mastectomy | 840 (39.1) | 486 (37.7) | 354 (41.3) |
|  | Other or unknown | 59 (3.2) | 32 (2.5) | 37 (4.3) |
|  | Missing | 28 | 14 | 14 |
| Comorbidities | 0 | 1318 (62.8) | 821 (65.5) | 497 (58.7) |
|  | 1 | 489 (23.3) | 290 (23.1) | 199 (23.5) |
|  | 2+ | 293 (13.9) | 142 (11.3) | 151 (17.8) |
|  | Missing | 75 | 50 | 25 |

**Supplementary Table 5:** Endocrine treatments within the high-risk subset according to the year of primary diagnosis (2016–2019 versus 2022–2023). [AI: aromatase inhibitor, TAM: tamoxifen, OFS: ovarian function suppression]

| **Characteristic** |  | **All patients (N=2175)** | **2016–2019 (N=1303)** | **2022–2023 (N=872)** |
| --- | --- | --- | --- | --- |
| Endocrine therapy as recommended | AI+OFS | 529 (25.2) | 119 (9.4) | 410 (49.0) |
|  | TAM monotherapy | 1273 (60.5) | 1002 (79.1) | 271 (32.4) |
|  | TAM+OFS | 265 (12.6) | 123 (9.7) | 142 (17.0) |
|  | Other | 36 (1.7) | 22 (1.7) | 14 (1.7) |
|  | Missing | 72 | 37 | 35 |
| Endocrine therapy as received | AI+OFS for 5 years | 501 (26.1) | 112 (9.2) | 389 (55.7) |
|  | TAM monotherapy for 5 years | 1193 (62.1) | 901 (73.7) | 292 (41.8) |
|  | AI+OFS followed by TAM±OFS | 16 (0.8) | 13 (1.1) | 3 (0.4) |
|  | TAM±OFS followed by AI+OFS | 195 (10.2) | 185 (15.1) | 10 (1.4) |
|  | Other | 15 (0.8) | 11 (0.9) | 4 (0.6) |
|  | Missing | 255 | 81 | 174 |

**Supplementary Table 6:** Patient characteristics of the high-risk population according to the recommended therapy. Patients for whom “other endocrine therapy” was recommended (N=36) and patients with missing information (N=72) were omitted from the analyses. [BMI: body mass index, SD: standard deviation, ECOG: Eastern Cooperative Oncology Group performance status, N: lymph nodes, T: tumor size, BET: breast conservation therapy, AI: aromatase inhibitor, TAM: tamoxifen, OFS: ovarian function suppression]

| **Characteristic** |  | **AI+OFS  (N=529)** | **TAM monotherapy  (N=1273)** | **TAM+OFS (N=265)** |
| --- | --- | --- | --- | --- |
| Age at initial diagnosis (years) | Mean (SD) | 43.8 (6.2) | 45.3 (5.5) | 40.5 (6.4) |
| BMI (kg/m^2^) | Mean (SD) | 25.8 (5.4) | 25.7 (5.2) | 25.0 (4.9) |
| ECOG | 0 | 392 (95.1) | 796 (95.6) | 198 (95.2) |
|  | 1–4 | 20 (4.9) | 37 (4.4) | 10 (4.8) |
|  | Missing | 117 | 440 | 57 |
| Ki-67 | <20% | 175 (33.7) | 478 (39.2) | 109 (41.6) |
|  | ≥20% | 345 (66.3) | 742 (60.8) | 153 (58.4) |
|  | Missing | 9 | 53 | 3 |
| Tumor grading | G1 | 29 (5.6) | 128 (10.3) | 27 (10.3) |
|  | G2 | 339 (65.1) | 795 (64.1) | 158 (60.1) |
|  | G3 | 153 (29.4) | 317 (25.6) | 78 (29.7) |
|  | Missing | 8 | 33 | 2 |
| Highest T | TX | 0 (0.0) | 0 (0.0) | 0 (0.0) |
|  | T0 | 5 (0.9) | 10 (0.8) | 2 (0.8) |
|  | Tis | 0 (0.0) | 5 (0.4) | 2 (0.8) |
|  | T1 | 87 (16.4) | 322 (25.3) | 56 (21.1) |
|  | T2 | 318 (60.1) | 762 (59.9) | 169 (63.8) |
|  | T3 | 91 (17.2) | 145 (11.4) | 30 (11.3) |
|  | T4 | 28 (5.3) | 29 (2.3) | 6 (2.3) |
|  | Missing | 0 | 0 | 0 |
| Highest N status | Nx | 0 (0.0) | 0 (0.0) | 0 (0.0) |
|  | N0 | 98 (18.5) | 364 (28.6) | 62 (23.4) |
|  | N1 | 285 (53.9) | 721 (56.6) | 160 (60.4) |
|  | N>1 | 146 (27.6) | 188 (14.8) | 43 (16.2) |
|  | Missing | 0 | 0 | 0 |
| Breast cancer anatomic stage | IIA | 139 (26.3) | 587 (46.1) | 109 (41.1) |
|  | IIB | 183 (34.6) | 419 (32.9) | 95 (35.8) |
|  | IIIA | 152 (28.7) | 201 (15.8) | 51 (19.2) |
|  | IIIB | 27 (5.1) | 28 (2.2) | 5 (1.9) |
|  | IIIC | 28 (5.3) | 38 (3.0) | 5 (1.9) |
|  | Not determinable | 0 (0.0) | 0 (0.0) | 0 (0.0) |
|  | Missing | 0 | 0 | 0 |
| Previous chemotherapy | Yes | 419 (79.2) | 948 (74.5) | 208 (78.5) |
|  | No | 110 (20.8) | 324 (25.5) | 57 (21.5) |
|  | Missing | 0 | 1 | 0 |
| Neoadjuvant chemotherapy | Yes | 270 (51.0) | 420 (33.0) | 115 (43.4) |
|  | No | 259 (49.0) | 853 (67.0) | 150 (56.6) |
|  | Missing | 0 | 0 | 0 |
| Adjuvant chemotherapy | Yes | 187 (35.3) | 575 (45.2) | 105 (39.6) |
|  | No | 342 (64.7) | 698 (54.8) | 160 (60.4) |
|  | Missing | 0 | 0 | 0 |
| Previous radiotherapy | Yes | 421 (79.9) | 1023 (80.9) | 212 (80.3) |
|  | No | 106 (20.1) | 242 (19.1) | 52 (19.7) |
|  | Missing | 2 | 8 | 1 |
| Type of surgery | BET | 262 (50.3) | 767 (60.9) | 142 (54.0) |
|  | Mastectomy | 242 (46.4) | 451 (35.8) | 114 (43.3) |
|  | Other | 17 (3.3) | 38 (3.0) | 6 (2.3) |
|  | Unknown | 0 (0.0) | 3 (0.2) | 1 (0.4) |
|  | Missing | 8 | 14 | 2 |
| Comorbidities | 0 | 291 (56.9) | 790 (64.4) | 170 (65.9) |
|  | 1 | 132 (25.8) | 279 (22.8) | 59 (22.9) |
|  | 2+ | 88 (17.2) | 157 (12.8) | 29 (11.2) |
|  | Missing | 18 | 47 | 7 |

**Supplementary Table 7:** Patient characteristics according to the recommended therapy for the patients from the overall population diagnosed in 2016–2019. Patients for whom “other endocrine therapy” was recommended (N=25) were omitted from the analyses. [BMI: body mass index, SD: standard deviation, ECOG: Eastern Cooperative Oncology Group performance status, N: lymph nodes, T: tumor size, BET: breast conservation therapy, CDK4/6i: CDK4/6 inhibitor, AI: aromatase inhibitor, TAM: tamoxifen, OFS: ovarian function suppression]

| **Characteristic** |  | **AI+OFS  (N=145)** | **TAM monotherapy**  **(N=1387)** | **TAM+OFS (N=160)** |
| --- | --- | --- | --- | --- |
| Age at initial diagnosis (years) | Mean (SD) | 43.7 (7.8) | 45.4 (5.5) | 38.4 (6.4) |
| BMI (kg/m^2^) | Mean (SD) | 25.5 (4.9) | 25.5 (5.1) | 25.1 (4.8) |
| ECOG | 0 | 77 (89.5) | 844 (95.8) | 105 (95.5) |
|  | 1–4 | 9 (10.5) | 37 (4.2) | 5 (4.5) |
|  | Missing | 59 | 506 | 50 |
| Ki-67 | <20% | 44 (32.4) | 600 (45.6) | 56 (35.7) |
|  | ≥20% | 92 (67.6) | 716 (54.4) | 101 (64.3) |
|  | Missing | 9 | 71 | 3 |
| Tumor grading | G1 | 6 (4.4) | 175 (13.1) | 17 (10.8) |
|  | G2 | 84 (61.3) | 824 (61.8) | 88 (55.7) |
|  | G3 | 47 (34.3) | 334 (25.1) | 53 (33.5) |
|  | Missing | 8 | 54 | 2 |
| Highest T | TX | 0 (0.0) | 2 (0.1) | 0 (0.0) |
|  | T0 | 5 (3.5) | 10 (0.7) | 3 (1.9) |
|  | Tis | 0 (0.0) | 13 (1.0) | 1 (0.6) |
|  | T1 | 38 (26.6) | 427 (31.3) | 51 (32.9) |
|  | T2 | 78 (54.5) | 782 (57.3) | 81 (52.3) |
|  | T3 | 17 (11.9) | 108 (7.9) | 16 (10.3) |
|  | T4 | 5 (3.5) | 22 (1.6) | 3 (1.9) |
|  | Missing | 2 | 23 | 5 |
| Highest N status | Nx | 0 (0.0) | 2 (0.2) | 0 (0.0) |
|  | N0 | 43 (30.3) | 607 (45.9) | 52 (34.2) |
|  | N1 | 66 (46.5) | 556 (42.1) | 77 (50.7) |
|  | N>1 | 33 (23.2) | 157 (11.9) | 23 (15.1) |
|  | Missing | 3 | 65 | 8 |
| Breast cancer anatomic stage | 0 | 3 (2.1) | 9 (0.6) | 1 (0.6) |
|  | IA | 13 (9.0) | 144 (10.4) | 18 (11.2) |
|  | IB | 4 (2.8) | 42 (3.0) | 5 (3.1) |
|  | IIA without CDK4/6i indication | 8 (5.5) | 177 (12.7) | 8 (5.0) |
|  | IIA with CDK4/6i indication | 33 (22.8) | 438 (31.6) | 46 (28.8) |
|  | IIB | 39 (26.9) | 297 (21.4) | 43 (26.9) |
|  | IIIA | 34 (23.4) | 161 (11.6) | 26 (16.2) |
|  | IIIB | 5 (3.4) | 20 (1.4) | 3 (1.9) |
|  | IIIC | 3 (2.1) | 31 (2.2) | 2 (1.2) |
|  | Not determinable | 3 (2.1) | 68 (4.9) | 8 (5.0) |
|  | Missing | 0 | 0 | 0 |
| Previous chemotherapy | Yes | 118 (81.9) | 922 (66.5) | 130 (81.2) |
|  | No | 26 (18.1) | 464 (33.5) | 30 (18.8) |
|  | Missing | 1 | 1 | 0 |
| Neoadjuvant chemotherapy | Yes | 74 (51.0) | 415 (29.9) | 89 (55.6) |
|  | No | 71 (49.0) | 972 (70.1) | 71 (44.4) |
|  | Missing | 0 | 0 | 0 |
| Adjuvant chemotherapy | Yes | 54 (37.2) | 548 (39.5) | 48 (30.0) |
|  | No | 91 (62.8) | 839 (60.5) | 112 (70.0) |
|  | Missing | 0 | 0 | 0 |
| Previous radiotherapy | Yes | 117 (81.2) | 1108 (80.2) | 127 (79.4) |
|  | No | 27 (18.8) | 273 (19.8) | 33 (20.6) |
|  | Missing | 1 | 6 | 0 |
| Type of surgery | BET | 82 (58.2) | 908 (66.5) | 91 (57.2) |
|  | Mastectomy | 58 (41.1) | 433 (31.7) | 65 (40.9) |
|  | Other | 1 (0.7) | 24 (1.8) | 3 (1.9) |
|  | Unknown | 0 (0.0) | 1 (0.1) | 0 (0.0) |
|  | Missing | 4 | 21 | 1 |
| Comorbidities | 0 | 88 (62.4) | 854 (63.6) | 102 (65.4) |
|  | 1 | 30 (21.3) | 326 (24.3) | 32 (20.5) |
|  | 2+ | 25 (16.3) | 163 (12.1) | 23 (14.1) |
|  | Missing | 4 | 44 | 4 |

**Supplementary Table 8**: Patient characteristics according to the recommended therapy for the patients from the overall population diagnosed in 2022–2023. Patients for whom “other endocrine therapy” was recommended (N=20) were omitted from the analyses. [BMI: body mass index, SD: standard deviation, ECOG: Eastern Cooperative Oncology Group performance status, N: lymph nodes, T: tumor size, BET: breast conservation therapy, CDK4/6i: CDK4/6 inhibitor, AI: aromatase inhibitor, TAM: tamoxifen, OFS: ovarian function suppression]

| **Characteristic** |  | **AI+OFS  (N=451)** | **TAM monotherapy (N=424)** | **TAM+OFS (N=177)** |
| --- | --- | --- | --- | --- |
| Age at initial diagnosis (years) | Mean (SD) | 44.0 (6.2) | 45.5 (5.4) | 42.0 (6.0) |
| BMI (kg/m^2^) | Mean (SD) | 26.0 (5.6) | 25.9 (5.3) | 25.2 (5.5) |
| ECOG | 0 | 357 (96.0) | 309 (96.0) | 144 (94.7) |
|  | 1–4 | 15 (14.0) | 13 (4.0) | 8 (5.3) |
|  | Missing | 79 | 102 | 25 |
| Ki-67 | <20% | 168 (37.4) | 230 (55.6) | 82 (46.6) |
|  | ≥20% | 281 (62.6) | 184 (44.4) | 94 (53.4) |
|  | Missing | 2 | 10 | 1 |
| Tumor grading | G1 | 36 (8.1) | 80 (19.6) | 21 (12.1) |
|  | G2 | 289 (64.8) | 263 (64.3) | 107 (61.8) |
|  | G3 | 121 (27.1) | 66 (16.1) | 45 (26.0) |
|  | Missing | 5 | 15 | 4 |
| Highest T | T0 | 3 (0.7) | 2 (0.5) | 2 (1.1) |
|  | Tis | 0 (0.0) | 2 (0.5) | 1 (0.6) |
|  | T1 | 84 (18.7) | 118 (28.0) | 52 (29.4) |
|  | T2 | 270 (60.1) | 256 (60.8) | 108 (61.0) |
|  | T3 | 70 (15.6) | 36 (8.6) | 13 (7.3) |
|  | T4 | 22 (4.9) | 7 (1.7) | 1 (0.6) |
|  | Missing | 2 | 3 | 0 |
| Highest N status | Nx | 0 (0.0) | 1 (0.2) | 0 (0.0) |
|  | N0 | 120 (26.9) | 220 (52.6) | 73 (41.2) |
|  | N1 | 218 (48.9) | 180 (43.1) | 90 (50.8) |
|  | N>1 | 108 (24.2) | 17 (4.1) | 14 (7.9) |
|  | Missing | 5 | 6 | 0 |
| Breast cancer anatomic stage | 0 | 0 (0.0) | 1 (0.2) | 2 (1.1) |
|  | IA | 15 (3.3) | 41 (9.7) | 20 (11.3) |
|  | IB | 6 (1.3) | 10 (2.4) | 6 (3.4) |
|  | IIA without CDK4/6i indication | 29 (6.4) | 104 (24.5) | 16 (9.1) |
|  | IIA with CDK4/6i indication | 102 (22.6) | 125 (29.5) | 59 (33.3) |
|  | IIB | 137 (30.4) | 96 (22.6) | 50 (28.2) |
|  | IIIA | 114 (25.3) | 28 (6.6) | 22 (12.4) |
|  | IIIB | 21 (4.7) | 7 (1.7) | 1 (0.6) |
|  | IIIC | 22 (4.9) | 5 (1.2) | 1 (0.6) |
|  | Not determinable | 5 (1.1) | 7 (1.7) | 0 (0.0) |
|  | Missing | 0 | 0 | 0 |
| Previous chemotherapy | Yes | 328 (72.7) | 219 (51.7) | 126 (71.2) |
|  | No | 123 (27.3) | 205 (48.3) | 51 (28.8) |
|  | Missing | 0 | 0 | 0 |
| Neoadjuvant chemotherapy | Yes | 214 (47.5) | 99 (23.3) | 60 (33.9) |
|  | No | 237 (52.5) | 325 (76.7) | 117 (66.1) |
|  | Missing | 0 | 0 | 0 |
| Adjuvant chemotherapy | Yes | 145 (32.2) | 127 (30.0) | 71 (40.1) |
|  | No | 306 (67.8) | 297 (70.0) | 106 (59.9) |
|  | Missing | 0 | 0 | 0 |
| Previous radiotherapy | Yes | 348 (77.5) | 303 (71.5) | 134 (76.1) |
|  | No | 101 (22.5) | 121 (28.5) | 42 (23.9) |
|  | Missing | 2 | 0 | 1 |
| Type of surgery | BET | 225 (50.9) | 256 (61.5) | 100 (57.1) |
|  | Mastectomy | 200 (45.2) | 137 (32.9) | 71 (40.6) |
|  | Other | 17 (3.8) | 21 (5.0) | 3 (1.7) |
|  | Unknown | 0 (0.0) | 2 (0.5) | 1 (0.6) |
|  | Missing | 9 | 8 | 2 |
| Comorbidities | 0 | 239 (54.8) | 254 (61.2) | 113 (65.3) |
|  | 1 | 120 (27.5) | 83 (20.0) | 42 (24.3) |
|  | 2+ | 77 (17.7) | 78 (18.8) | 18 (10.4) |
|  | Missing | 15 | 9 | 4 |
